# Dairy products and colorectal cancer in middle eastern and north African countries: a systematic review

**DOI:** 10.1186/s12885-018-4139-6

**Published:** 2018-03-01

**Authors:** K. El kinany, M. Deoula, Z. Hatime, B. Bennani, K. El Rhazi

**Affiliations:** 1 Department of Epidemiology and Public Health, Faculty of Medicine and pharmacy of Fez, Sidi Mohamed Ben Abdellah University, Fez, Morocco; 2 Faculty of Science Dhar Mehraz, Laboratory of Microbiology and Molecular Biology, Fez, Morocco; 3 Laboratory of Microbiology and Molecular Biology, Faculty of Medicine and Pharmacy of Fez, Sidi Mohamed Ben Abdellah University, Fez, Morocco

**Keywords:** Dairy products, Colorectal cancer, Risk, Prevention, Middle eastern and north African countries, Systematic review

## Abstract

**Background:**

This systematic review was conducted to explain the association between dairy products and colorectal cancer (CRC) risk in Middle Eastern and North African countries (MENA).

**Methods:**

The database consulted were PubMed, Clinical Trials, and Cochrane to extract the relevant studies published till the 31stof December 2016, using inclusion and exclusion criteria according to Prisma Protocol. The characteristics of these studies comprised the consumption of all types of dairy products in relation to CRC risk.

**Results:**

Seven studies were included in this review. For dairy products overall, no significant association was found. Regarding modern dairy products, included studies found controversial results with OR = 9.88 (95% CI: 3.80–24.65) and OR_a_ = 0.14 (95% CI: 0.02–0.71). A positive association was reported between traditional dairy products and CRC risk, to OR = 18.66 (95% CI: 3.06–113.86) to OR = 24 (95% CI: 1.74–330.82) to ORa = 1.42 (95% CI: 0.62–3.25), p_trend_ = 0.03. Calcium was inversely associated with the CRC risk with OR_a_ = 0.08 (95% CI: 0.04–0.17).

**Conclusion:**

This is the first systematic review which illustrated the association between dairy consumption and CRC risk in MENA region. The results were inconsistent and not always homogeneous. Further specified studies may be warranted to address the questions about the association between CRC and dairy products in a specific context of MENA region.

## Background

Colorectal cancer (CRC) is the third most commonly diagnosed cancer worldwide [1], with nearly 1.4 million new cases diagnosed in 2012 and 694.000 deaths [2]. There is a large geographical variation of CRC incidence, that is very high in developed countries compared with developing countries [3], but there is an increasing incidence in countries undergoing nutritional transitions [4, 5].

Several studies have provided solid evidence that lifestyle and dietary factors are likely to be the major determinants of CRC risk [6–12].

Milk and dairy products have the distinction of being composed of different elements; some of which could hypothetically increase the risk of certain diseases [13]; while others may decrease it [14]. In fact, the evidence that milk and calcium protect against CRC was judged as probable by an international panel of experts [12, 15, 16]. Most of these results come from North-American and European countries. Little is known about this relationship in MENA countries.

MENA countries have several common factors such as environment, culture, and some dietary habits. Furthermore, this region is incurring nutrition transition, which is associated with an increased burden of non-communicable diseases [17–19]. This nutrition transition is characterized by the increasing consumption of some westernized foods including dairy products [20].

There are two types of dairy products in this region: modern products which are similar to European countries as (total, semi-skimmed, and skimmed) milk, (hard, semi-hard, soft and fresh) cheese, and (double, fresh and ice) cream, and traditional products which differ by their composition. The main traditional dairy products of North African countries as well as in Middle East countries are Lben, Raib, Jben, Klila, zebda beldia, Zabadi, Karish cheese, Aoules, Tallaga cheese, Mish cheese, Domiati cheese, Rigouta, Kishk, Laban, Labaneh, Shenineh, Shenglish, Keshkeh, Akawieh, kefir and Chelal [21, 22]. All these traditional dairy products are prepared by simply allowing the raw milk to ferment spontaneously at room temperature (15° to 25 °C) for 1 to 3 days depending on the season [23]. The presence of mycotoxins, the lack of veterinary care, and the poor sanitary conditions are the biggest problems challenging public health safety of these products [21].

The consumption of dairy products in MENA region has increased during the last two decades from 30 to 150 kg/capita/year [24]. However, this increase is small when compared with the main producing countries such as India, the United States of America, China, Pakistan and Brazil [25].

The increasing incidence of CRC in this region could be related to this nutrition transition and also to the nutritional specificities of this region, including traditional dairy products which may affect the genetic mutation profile.

The present systematic review aimed at describing the associations between dairy products and CRC risk in MENA countries, based on the published scientific literature.

## Methods

### Search strategy

We conducted an exhaustive search for full text articles in databases, namely in: Pub Med (*http://www.****ncbi.nlm.nih****.gov*), Cochrane (*www.thecochranelibrary.com*), and in Clinical Trials (***clinicaltrials****.gov**)*. We used the key words “dairy products” (any type of Milk, whole milk, skimmed milk, semi skimmed milk, milk free fat, soya milk), Cheese (hard, soft, fresh, semi hard), Yogurt, Cream (ice cream, fresh cream, double cream), “traditional dairy products” (Lben, Raib, Jben, Klila, zebda beldia, Zabadi, Karish cheese, Aoules, Tallaga cheese, Mish cheese, Domiati cheese, Rigouta, Kishk, Labaneh, Shenineh, Shenglish, Keshkeh, Akawieh, and Chelal); and “Colorectal cancer, Colon cancer, and Rectal cancer”. We have also selected the areas of “*North African countries”* (Algeria, Egypt, Libya, Morocco, Sudan, and Tunisia) and “*Middle east countries”* (Turkey, Bahrain, Iraq, Iran, Israel, Jordan, Kuwait, Lebanon, Oman, Palestine, Qatar, Saudi Arabia, Syria, United Arab Emirates, and Yemen). All identified studies published until the 31^st^ December 2016 were considered.

### Inclusion criteria

The studies that were included in this review were original studies conducted among people living in the MENA region. The surveys investigated the associations between dairy products and CRC, and provided estimates of the associations, by reporting the odds ratio (OR) or relative risk (RR) for analytical studies or means comparison and differences in the percentage for clinical trials with 95% confidence intervals (CIs) or *p*-value. All reviewed articles were published in English or French. Ecological [26, 27], laboratory and animal [28–31] studies, and off topic studies [32–35] were excluded (Table 1). The bibliographic research took place over a period of two months.

**Table 1:** Characteristics of excluded studies

Author; date	Country	Type of study	Exclusion criteria
Abbastabar et al., (2015) [34].	Iran	Ecological study	Risk not specified.
Khoury et al., (2014) [39].	Lebanon	Experimental study.	Experimental Research in vitro using cell line and cell culture.
Rohani et al., (2013) [35].	Iran	Ecological study	Risk not specified.
Habib et al., (2013) [38].	United Arab Emirates	Experimental study.	Experimental research in vitro using culture of cell.
Attaallah et al., (2012) [36].	Turkey	Experimental study.	Experimental Research in vivo using rats.
Bener et al., (2010) [41].	Qatar	Case control study	Not examine the relationship between dairy products and CRC.
Almurshed et al., (2009) [40].	Saudi Arabia	Case control study	Not examine the relationship between dairy products and CRC.
Can et al., (2009) [42].	Turkey	Clinical trial	Study the quality of life in patients being treated for CRC.
Topuz et al., (2008) [43].	Turkey	Randomized prospective observational study	Examine the effect of oral kefir administration on serum pro-inflammatory cytokine levels in patients with CRC.
Cenesiz et al., (2008) [37].	Turkey	Experimental study.	Experimental research in vivo using mice.

### Extraction data

We extracted the following data in each paper intended for reviewing: the name of the first author, the country as well as the design of study, the number of participants and the year of publication, the exposure and confounding factors, the specific characteristics and the outcomes, the main findings and the effects.

**Fig. 1:**
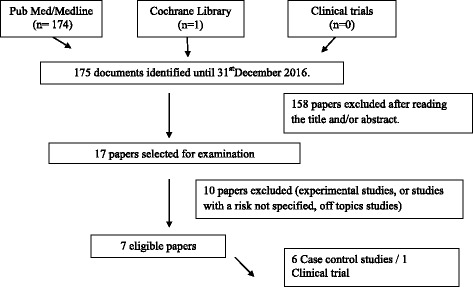
The PRISMA Diagram of the selected papers

17 Relevant publications were selected first upon reading their titles and abstracts, and by reading the full texts of the chosen articles. Upon excluding ten studies which did not meet the criteria (for the most part laboratory and animal studies), only seven studies were singled out for reviewing (Fig. 1).

### Quality assessment

The quality of the included studies was assessed using PRISMA guidelines [36], and they were evaluated by the following lines: the accuracy as well as the validity of the questions (answers per evidence), and the representability of the studied population. The synthesis (Table 2) reflected the strength of the findings in relation to the types of the study design [37] (level), and their methodological weaknesses (the biases and limitations of each study).

**Table 2:** Quality assessment of published papers on dairy products and CRC risk in the Middle East and North African countries

Author/Reference	Relevant to this SR	Aims clearly stated	Appropriate study method	Sample representative of target population	Confounding and bias considered	Good Responsse rate?	Were questions piloted / validated?	Tables/figures understandable	Can results be applied to local situation?	Accepted as Type IV evidence?
Tayyem et al., (2016) [50].	Yes	Yes	Yes	Yes	Yes	Yes	Yes	Yes	Yes	No, type (III)
Suhad et al., (2015) [47].	Yes	Yes	Yes	Yes	Yes	Yes	Yes	Yes	Yes	No, type (III)
Mahfouz et al., (2014) [51].	Yes	Yes	Yes	No	No	Yes	No	Yes	No	No, type (III)
Arafa et al., (2011) [46].	Yes	Yes	Yes	No	No	Yes	No	Yes	No	No, type (III)
Guesmi et al., (2010) [48].	Yes	Yes	No	No	No	Yes	No	Yes	No	No, type (III)
Nashar et al., (2008) [49].	Yes	Yes	Yes	No	No	Yes	No	Yes	No	No, type (III)
Rozen et al., (2001) [52].	Yes	Yes	Yes	No	Yes	No	Yes	Yes	No	No, type (II)

## Results

Seven studies were included in this review, representing five countries: Egypt, Jordan (Arafa et al., Suhad et al., and Tayyem et al.,), Israel, Saudi Arabia, and Tunisia. The study results were summarized in Table 3.

**Table 3:** Main results of Included Studies

Author/ Year/Reference	County and setting	Study design and Population	Exposure and Confounders	Outcome	Comparison	Main finding and effect
Tayyem et al., (2016) [50].	-Jordan -Five large Jordanian hospitals including oncology services.	-Case control study − 220 Cases were selected from five large Jordanian hospitals with oncology services. − 281 Controls were selected from hospital personnel, outpatients and visitors.	-Exposure: meats, dairy products and fat. -Confounders: age, sex, BMI, PA, total EI, income, occupation, education level, marital status, cigarette smoking, other health problems and family history of CRC	CRC in both sexes	-Group 1: CRC cases (116 males and 104 females). -Group 2: healthy disease-free controls (Number of males and females was not determined).	The daily consumption of: - Labaneh OR_a_ = 1.42 (95% CI: 0.62–3.25), p_trend_ = 0.03. -Milk OR_a_ = 1.24 (95% CI: 0.62–2.47), ptrend = 0.59. -Yoghurt OR_a_ = 0.76 (95% CI: 0.25–2.32), p_trend_ = 0.65. -White cheese OR_a_ = 1.06 (95% CI: 0.46–2.45), ptrend = 0.06. - Ice cream: OR_a_ = 1.68 (95% CI: 0.77–3.65), p_trend_ = 0.11. The weekly consumption of: - Cooked yogurt OR_a_ = 0.59 (95% CI: 0.26–1.39), p_trend_ = 0.03. The monthly consumption of: - Processed cheese OR_a_ = 0.29(95% CI: 0.06–1.45), p_trend_ = 0.004.
Suhad et al., (2015) [47].	-Jordan -Five large Jordanian hospitals including oncology services.	-Case control study − 167 Cases were selected from five major Jordanian hospitals including an oncology center. − 240 Controls were selected from hospital personnel, outpatients, visitors, and accompanying individuals (not a first degree relative).	-Exposure: five food groups-grains, vegetables, fruits, milk, and meat and legumes. -Confounders: total EI, age, sex, PA, family history of CRC, household income, marital status, and cigarette smoking.	CRC in both sexes	-Group 1: CRC cases (79 males and 88 females). -Group 2: Healthy controls (108 males and 132 females).	-Milk OR_a_ = 0.75(95% CI: 0.40–1.40), -Yoghurt OR_a_ = 0.621 (95% CI: 0.36–1.06) -Labaneh OR_a_ = 1.32 (95% CI: 0.76–2.27) -White cheese OR_a_ = 1.46 (95% CI: 0.86–2.47).
Mahfouz et al., (2014) [51].	-Egypt -Minia oncology center	-Case control study − 150 Cases were selected from Minia oncology center. − 300 Controls were selected from community	-Exposure: dietary and lifestyles factors. -Confounders: PA, fruit and vegetables.	CRC in both sexes	-Group 1: CRC cases receiving any treatment (72 males and 78 females) -Group 2: Controls (144 males and 156 females).	Inverse association with calcium rich diet OR_a_ = 0.08 (95% CI: 0.04–0.17).
Arafa et al., (2011) [46].	-Jordan -Al-Bashir Hospital, the principal governmental center for CRC registry and therapy	-Case control study − 220 Cases were selected from Al-Bashir Hospital. − 220 Controls were selected from the outpatient departments.	-Exposure: smoking, alcohol drinking, family history of CRC, vitamins supplement, monthly income and physical activity, dietary intake using a FFQ. -Confounders: routine exercise practice, smoking history, BMI, fruit, vegetables, meats, tea.	CRC in both sexes	Group 1: CRC cases (118 males and 102 females) Group 2: Controls (118 males and 102 females).	-Milk, yogurt and cheese group OR = 1.60 (95% CI: 0.84–3.04). -Calcium OR_a_ = 0.99 (95% CI: 0.99–1.00).
Guesmi et al., (2010) [48].	-Tunisia -Surgery service in Nicole Charles Hospital And in Institut Salah Azaiez of Cancerology	-Case control study − 32 Cases were selected from Nicole Charles Hospital And in Institut Salah Azaiez of Cancerology − 61 Controls were selected from surgery and orthopaedic departments.	-Exposure: alimentary factors like meats group, fruits, vegetables, Raw oil, olive oil, full cereals, sweets and methods of cooking. -Confounders: Age, sex, geographic origin, smoking, anemia, sport, walking, frequency of consumption (frequently / rarely), methods of cooking.	CRC in both sexes	-Group 1: CRC cases (12 males and 20 females) -Group 2: Controls (39 males and 22 females).	Milk OR_a_ = 0.14 (95% CI: 0.02–0.71).
Nashar et al., (2008) [49].	-Saudi –Arabia -King Faisal Specialist Hospital & Research Center, Riyadh	-Case-control study −50 Cases were selected from the inpatients in KFSH and RC. − 50 Controls were selected from the outpatients in KFSH and RC.	-Exposure: Eating habits with the frequency of consumption. - Confounders: frequency of consumption.	Newly colon cancer in both sexes	-Group 1: CRC cases (25 males and 25 females). -Group 2: healthy controls (25 males and 25 females).	-For both sexes -Milk OR = 9.88 (95% CI: 3.80–24.65). -Laban OR = 18.66 (95% CI: 3.06–113.86). -Labnah OR = 24 (95% CI: 1.74–330.82). -For men -Cheese OR = 8 (95% CI: 1.40–45.75). -Laban OR = 15 (95% CI: 1.58–142.17). -For females -Laban OR = 27 (95% CI: 1.26–578.38).
Rozen et al., (2001) [52].	-Israël -Gastro-enterology department at the Tel Aviv Medical Center	-Clinical trial − 125 adenoma patients: − 68 Intervened patients receiving 1.5 g calcium ion/day (5 chewable calcium carbonate tablets daily). − 57 Non-intervened patients receiving no treatment.	-Exposure: dietary factors, lifestyle habits, and calcium supplementation. -Confounders: dietary components as fat, carbohydrates, fiber, and fluid as well as tobacco uses.	Adenoma patients, without a family history of colorectal neoplasia.	- Intervention group: 33 patients completed the 1 year trial (20 males and 13 females). - Non-intervention group: 19 patients completed rectal biopsy (13 males and 6 females).	-The REP labeling index decreased in 58% of calcium-intervened patients and in only 26% of non-intervened patients (*p* = 0.04). -The interaction between the mean daily total fat and calcium effect on LI was significantly negative (in opposite directions *p* = 0.02). -The interaction between the higher mean daily intake of total carbohydrates and the effect of calcium on LI was significantly positive (*p* = 0.001)

EI: energy intake; CI: confidence interval; FFQ: food frequency questionnaire, OR^a^: Adjusted Odds Ratio; REP: rectal epithelial proliferation; LI: labeling index; BMI: body mass index; PA: physical activity.

Concerning the relation between overall dairy products (milk, yogurt, cheese, and Labaneh) and CRC risk, the Jordanian studies (Arafa et al., and Suhad et al.,) [38, 39] did not find any significant association.

Regarding modern dairy products, the Tunisian and the Saudi Arabian studies [40, 41] found controversial results. The Saudi Arabian study found an increased risk of CRC related to milk OR = 9.88 (95% CI: 3.80–24.65), while the Tunisian study found a decreased risk of CRC related to milk OR = 0.14 (95% CI: 0.02–0.71). Concerning cheese consumption, the Saudi Arabian study [41] found it a risk factor OR = 8 (95% CI: 1.40–45.75) only for men.

As for traditional dairy products and CRC risk, the Saudi Arabian and the Jordanian studies [41, 42] demonstrated that traditional dairy products were a risk factor. For a Jordanian study (Tayyem et al.,) [42], the consumption of labaneh was found to be associated with the risk of CRC (OR = 1.42, P_trend_ = 0.038), likewise the Saudi Arabian study [41] showed that the consumption of laban, and labaneh, 4 times or above a week resulted in an increase in the CRC risk respectively Laban OR = 18.66 (95% CI: 3.06–113.86) and Labnah OR = 24 (95% CI: 1.74–330.82).

For the relationship between calcium and CRC risk, the Egyptian [43] and Israelian [44] studies found that calcium is a protective factor. For the Egyptian study, calcium rich diet was considered as a protective factor with OR = 0.08 (95% CI: 0.04–0.17). The Israelian clinical trial concluded that long-term calcium supplements and long-term dietary habits significantly suppressed rectal epithelial proliferation (REP) in adenoma patients.

## Discussion

This systematic review aimed at describing the relationship between dairy products and CRC in MENA countries. Some of these included studies reported that dairy products were a protective factor for CRC; others considered them as a risk factor.

Three studies in total found that dairy products were protective factors, representing three countries in this region: Egypt, Tunisia, and Israel. Several studies found similar results and showed that milk was considered as a protective factor because of its high calcium concentration [45–49]. In fact, the high intake of calcium was associated with a decreased risk for CRC [50] and calcium supplements were used to prevent CRC [51]. Moreover, milk constituents other than calcium may also contribute to the anti-neoplastic activity, including conjugated linoleic acid (CLA) which has antioxidant, anti-inflammatory and immune modulatory properties [52–54].

Saudi Arabian, and Jordanian studies [41, 42] found that dairy products including traditional ones were considered as risk factors. This result was similar to a longitudinal study which concluded that highly childhood dairy intake increased CRC risk [55]. For traditional dairy products, despite the acidic nature of these products (pH 5.0–5.5) [22] they showed a high number of indicator microorganisms [56]. This can be explained by the poor hygienic conditions in which these products were prepared, as well as the poor bacteriological quality of the raw milk used for their manufacture [22]. Furthermore, these traditional products are high in fat content [57]. Several studies showed that a high fat consumption increased the concentration of bile acid which can promote CRC [58–60].

In the same country Jordan, two case-control studies (Arafa et al., and Suhad et al.,) [38, 39] did not find any relationship between dairy products and the risk of CRC development. Some cohort studies showed the same results but only for total milk [61].

The results of the examined surveys are not only inconsistent and controversial, they have in addition several limitations: Some studies were conducted based on a small sample size and the controls were recruited among inpatients [40, 41] who have other diseases than cancer and have been following a diet because of them. Thus, these samples may not be representative of the targeted population.

Regarding the Egyptian study [43], it included already treated cases of CRC, which may affect the quality of the collected data in the way that patients probably, changed their diet after being diagnosed. Indeed, the study did not exclude cases and controls that followed a diet.

Moreover, dietary history was evaluated in most of these studies, by the Food Frequency Questionnaire (FFQ) and during 2 years earlier to cancer as it is the case for the Egyptian study. In most cases, these FFQs were not validated and the frequency of each food consumption was calculated by a scale of two values: Rare /frequent. Thus, the quality of usable questionnaire was weak which might have led to a lack of information and precision, and might have over- or under-estimated dietary intake.

Equally important, data analysis was not always adjusted for all potential confounders as energy intake, BMI, nutrient intake, and alcohol intake. Therefore, results from these studies ought to be interpreted with caution.

The major limit of the Israelian study [44], even if it’s a prospective study, was the low number of voluntary participants, alongside with the large proportion of intervened patients who did not finish the 1 year of calcium intervention and non-intervened patients who did not comply with the 1 year rectal biopsy. This study may lack of power and its results may not apply in a similar situation.

## Conclusion

This review, which is the first study in its kind in MENA countries, presented the main results about the association between CRC and dairy products in this region. The highlighted results were inconsistent, controversial, and studies had several limitations. Further studies with a best quality of methodology, are needed to address the questions about the association between CRC and dairy products in a specific context of MENA region.

## Abbreviations

CIs: Confidence Intervals
CLA: Conjugated Linoleic Acid
CRC: Colorectal cancer
FFQ: Food Frequency Questionnaire
MENA: Middle Eastern and North African countries
OR: Odds Ratio
REP: Rectal Epithelial Proliferation
RR: Relative Risk
